# Adherence to Statin Therapy and Attainment of LDL Cholesterol Targets in an Outpatient Population of Type 2 Diabetes Patients: Analysis in the DIAbetes and LifEstyle Cohort Twente (DIALECT)

**DOI:** 10.3389/fphar.2022.888110

**Published:** 2022-07-12

**Authors:** Jelle M. Beernink, Milou M. Oosterwijk, Job F. M. van Boven, Hiddo J. L. Heerspink, Stephan J. L. Bakker, Gerjan Navis, Roos M. Nijboer, Christina M. Gant, Heleen Haverkate, Willemien J. Kruik-Kollöffel, Gozewijn D. Laverman

**Affiliations:** ^1^ Department of Internal Medicine/Nephrology, Ziekenhuis Groep Twente, Almelo, Netherlands; ^2^ Department of Clinical Pharmacy and Pharmacology, University Medical Center Groningen, University of Groningen, Groningen, Netherlands; ^3^ Medication Adherence Expertise Center of the Northern Netherlands (MAECON), Groningen, Netherlands; ^4^ Division of Nephrology, Department of Internal Medicine, University of Groningen, University Medical Center Groningen, Groningen, Netherlands; ^5^ Department of Internal Medicine, Meander Medical Center, Amersfoort, Netherlands; ^6^ Department of Clinical Pharmacy, Ziekenhuis Groep Twente, Almelo, Netherlands; ^7^ Biomedical Signals and Systems (BSS), University of Twente, Enschede, Netherlands

**Keywords:** diabet mellitus type 2, statin (HMG-CoA reductase inhibitor), medication adherance, medication possesion ratio, LDL—cholesterol, cholesterol, lipid lowering medication, LDL cholesterol targets

## Abstract

**Objective:** To assess adherence to statin therapy and its association with sociodemographic data, medical characteristics, LDLc levels, and LDLc target attainment in real-world T2D patients treated in secondary care.

**Research Design and Methods:** Cross-sectional analyses were performed on baseline data of 393 patients in the DIAbetes and LifEstyle Cohort Twente (DIALECT). The medication possession ratio (MPR), calculated with pharmacy dispensing data, was used to determine adherence to statins for an intended period of 24 months. Statins were included in the analyses if they were used for at least six consecutive months with at least three dispenses. Adherence was defined as an MPR ≥80%. Associations with adherence were assessed using descriptive statistics and binary logistic regression.

**Results:** Overall, 80% of the patients had a statin prescription and of those, 89% were adherent. The proportion of patients who reached LDLc targets of ≤2.5 mmol/L and <1.8 mmol/L differed significantly between the adherent, nonadherent and non-statin group (90% vs. 74% vs. 46%; *p* < 0.01 and 56% vs. 26% vs. 6%; *p* < 0.01, respectively). Serum LDLc levels were lower in the adherent versus the nonadherent and non-statin group (1.76 ± 0.60 vs. 2.23 ± 0.90 vs. 2.71 ± 0.67 mmol/L; *p* < 0.01). Higher HbA1c levels were independently associated with nonadherence (OR: 1.05, 95% CI 1.01–1.08; *p* < 0.01). Mediation adherence (OR: 2.88, 95% CI 1.04–7.97; *p* = 0.041) and lower BMI (OR: 0.88, 95% CI 0.81–0.96; *p* < 0.01) were independently associated with attaining the LDLc target of ≤2.5 mmol/L.

**Conclusion:** In patients with T2D treated in secondary care, statin adherence was relatively high and was associated with significantly lower LDLc levels. It is important to identify nonadherence as it appeared an important determinant of failure to reach LDLc targets. The finding that many patients who failed to attain LDLc targets did not receive statin treatment offers an opportunity to improve diabetes care.

## Introduction

Type 2 diabetes (T2D) is associated with an increased risk for cardiovascular complications (Emerging Risk Factors Collaboration Sarwar et al., 2012; Rana et al., 2016). Prevention of cardiovascular complications by treatment of dyslipidaemia is therefore one of the main goals of diabetes care. Indeed, lowering of low-density lipoprotein cholesterol (LDLc) in T2D consistently reduces cardiovascular events (Colhoun et al., 2004; De Vries et al., 2012; De Vries et al., 2014; Burggraaf and Castro Cabezas, 2017). Given the strong association between LDLc and cardiovascular outcomes, diabetes guidelines provide treatment recommendations in order to reach specific LDLc targets (Piepoli et al., 2016; Kwaliteitsinstituut voor de Gezondheidszorg CBO, Nederlands Huisartsen Genootschap, 2020). Nevertheless, a recent Dutch study in the Diabetes and LifEstyle Cohort Twente (DIALECT) showed that the LDLc target of ≤2.5 mmol/L was not achieved by approximately 25% of this real-world cohort of patients with long-standing complicated T2D (Gant et al., 2018).

To improve long-term clinical outcomes, it is important to identify causes for failure of reaching LDLc treatment targets, especially in those with a very high cardiovascular risk profile. Notably, patient adherence to lipid-lowering drugs is a key factor to take into account. Previous studies have shown high rates of nonadherence to statin therapy (17.8–79.2%) (Hope et al., 2019). However, the majority of these studies did not assess the association of adherence with LDLc levels and LDLc target attainment, and some of these studies did assess adherence using patient self-report questionnaires, which might have resulted in over- or under-reporting (Perreault et al., 2009; Stuurman-Bieze et al., 2013; Wallach-Kildemoes et al., 2013; Halava et al., 2014; Farsaei et al., 2015). We aim to assess adherence to statin therapy using pharmacy dispensing data and its association with sociodemographic data, medical characteristics, LDLc levels and LDLc target attainment in a group of 393 real-world patients with complicated T2D.

## Materials and Methods

### Study Design and Setting

This study was performed in the DIAbetes and LifEstyle Cohort Twente-1 (DIALECT-1) cohort (Gant et al., 2017). DIALECT is an observational prospective cohort study performed in the Ziekenhuis Groep Twente Hospital (Almelo and Hengelo, Netherlands) and designed to investigate the effect of lifestyle and dietary habits and pharmacological treatment on outcomes in patients with complicated T2D treated in secondary care. The primary aim of DIALECT is to identify targets for the improvement of treatment quality by a systematic assessment of both pharmacological and nutritional management. Patients in the DIALECT-1 population were recruited between September 2009 and January 2016 (*n* = 450). Our study was performed according to the guidelines of good clinical practice and the declaration of Helsinki. Written informed consent was obtained from all patients before participation. The study has been approved by the local institutional review boards (Medisch Ethische Toetsingscommissie Reg. Nos., NL57219.044.16 and 1009.68020) and is registered in Netherlands Trial Register (NTR trial code 5855).

### Participants

The study population consisted of patients with T2D aged ≥18 years treated in the outpatient clinic as part of routine secondary care. In Netherlands, criteria for referral from primary to secondary health care are inability to achieve adequate glycaemic control [defined as failure to achieve the HbA1c target, which is usually ≤7% (53 mmol/mol)] with oral antidiabetics or a standard insulin regimen, macroalbuminuria and/or estimated glomerular filtration rate ≤60 ml/min, or multiple cardiovascular complications (Gant et al., 2018). Patients on renal replacement therapy or patients with insufficient knowledge of the Dutch language were excluded from participation.

Eligible patients were selected from the electronic patient file and contacted by phone, as described in detail previously (Gant et al., 2017). Of the original 450 patients included in DIALECT-1, 15 patients were excluded at a later stage because it turned out their actual diagnosis was type 1 diabetes (*n* = 9) or LADA (*n* = 2). Other reasons were dialysis before inclusion (*n* = 1) or because patients were included in the database twice (*n* = 2). Of these 435 patients, 393 patients were eligible for the current study. We excluded patients who did not have a baseline LDL laboratory value (*n* = 9), those with no informed consent for collecting pharmacy data (*n* = 17), those from whom no pharmacy data were available (*n* = 13), and those intolerant to statins due to side-effects (*n* = 3). Characteristics of excluded patients did not differ materially from those who were eligible for the current study (Supplementary Table S1).

### Baseline Demographics and Clinical Variables

At the outpatient clinic, baseline sociodemographic characteristics, medical history, lifestyle behaviours, and current medications were recorded. Anthropometric dimensions were measured using standard procedures. Non-fasting blood tests were taken at baseline visit to determine serum LDLc, total cholesterol, and HbA1c. Further details concerning baseline demographics and clinical variables have been described previously (Gant et al., 2017).

### Measurement of Adherence to Statins

For this study, pharmacy dispensing data were used to determine medication adherence. All the patients included in this study were re-approached in 2016 and 2017 in order to obtain new informed consent for collecting pharmacy data. Pharmacies were subsequently approached to provide the complete medication dispensing history of the patient from the baseline date of DIALECT-1 up to that day. As for the loss of patients, all the patients included in this study are under long-term treatment in our hospital. For patients who were referred to primary care or for patients who moved to another location, we had access to their contact details, which allowed us to approach them to provide consent for collecting pharmacy data. Analysis of the medication dispensing history was performed for an intended period of 24 months starting from the baseline visit. Using the pharmacy dispensing data, we calculated the number of tablets every patient obtained for each individual chronic medication during the intended 24-months follow-up. For each chronic medication, the first dispensing date after baseline and corresponding data about the number of tablets and dose were noted. The end date was defined as the date of the day before the last collection. Statins were included in the analyses if they were used for at least six consecutive months with at least three dispenses.

Adherence was subsequently determined by calculating the medication possession ratio (MPR), an adequate and well-accepted proxy for medication adherence by using pharmacy dispensing data (Steiner and Prochazka, 1997). The MPR is the proportion of time that prescribed medication is actually available for the patient and is defined as the ratio between the sum of days’ supply for all fills in a certain period and the number of days in that period. Good adherence was defined as an MPR ≥80% (Anghel et al., 2019). By default, 26 of the included patients were provided an automated medication dispensing system (Baxter Healthcare Corporation, Deerfield, LI, United States). These patients had an MPR of 100%. Changes to another statin or dosage during the follow-up period were carefully documented in the database. Left over medication after a change to another statin or dosage was subtracted from the total number of pills and accordingly, left over medication was not included in analyses.

### Cholesterol Targets

We assessed the association of medication adherence with two common LDLc targets. The primary treatment target for LDLc was ≤2.5 mmol/L, in line with the Dutch guidelines for cardiovascular risk management in T2D (Kwaliteitsinstituut voor de Gezondheidszorg CBO, Nederlands Huisartsen Genootschap, 2020). In addition, we studied associations with the LDLc target of <1.8 mmol/L for patients with a very high risk of CVDs (97% of our population) that is advocated in the European guideline for CVD prevention (Piepoli et al., 20162016). Finally, we assessed associations with serum LDLc and total cholesterol levels.

### Other Clinical Outcomes

In addition to associations of adherence with cholesterol outcomes, we assessed associations with other intermediate clinical characteristics (e.g., diabetes duration, HbA1c, and blood pressure), and microvascular and macrovascular complications. Details concerning the intermediate clinical characteristics and definitions of microvascular and macrovascular complications have been described previously (Gant et al., 2017).

### Statin Type and Intensity

Associations of adherence with statin type (simvastatin, atorvastatin, rosuvastatin, fluvastatin, and pravastatin) and intensity were also tested. Three statin treatment intensities were defined: “medium intensity” statin treatment was defined as simvastatin 20–40 mg/day, atorvastatin 10–20 mg/day, rosuvastatin 5 mg/day, or pravastatin 40–80 mg/day (Helfand et al., 2006). Lower and higher prescribed dosages were defined as “low-intensity” and “high-intensity” statin treatment, respectively.

### Statistical Analysis

All statistical analyses were performed using IBM SPSS for Windows (version 24.0; IBM Corp., Armonk, NY, United States). Normally distributed data are presented as mean ± SDs. Skewed variables are presented as median [interquartile ranges (IQRs)]. Dichotomous variables are presented as number (percentages). A two tailed *p* value < 0.05 was considered statistically significant. Normality of data was assessed using the Kolmogorov-Smirnov and Shapiro-Wilk tests of normality and by visually inspecting the frequency histograms of each variable. Post Hoc Tukey’s range tests were performed to assess if any of the three groups were statistically significantly different from each other. Significant differences determined by the Tukey’s range test are indicated by an asterisk (*). If all groups differed statistically significantly from each other, the asterisk was omitted.

The population was divided into two groups according to their adherence based on pharmacy dispensing data (MPR ≥80% or MPR <80%) and a third group consisting of patients without statin prescription. Differences between the adherent, nonadherent and non-statin group in sociodemographic data, medical characteristics, LDLc levels, and LDLc target attainment (≤2.5 mmol/L and <1.8 mmol/L) were tested using the one-way analysis of variance for normally distributed variables, Kruskal-Wallis for skewed variables, and the χ^2^ test for dichotomous variables. Determinants of nonadherence and determinants of attaining the LDLc target of ≤2.5 mmol/L were studied using binary logistic regression analysis based on complete cases. Potential confounders were selected based on relevant differences in characteristics in the baseline table, biological plausibility and previous literature.

## Results

### Descriptive Data

The mean age was 63 ± 9 years (Table 1), median diabetes duration was 11 (7–18) years, mean HbA1c was 57 ± 12 mmol/mol (7.4 ± 3.2%), and the mean BMI was 33 ± 6 kg/m^2^, reflecting a population with advanced T2D.

**TABLE 1:** Baseline characteristics by overall adherence in the DIALECT-1 population.

	Total population	Adherent	Nonadherent	No statin	*p*
Patients	393	280 (89.2)	34 (10.8)	79 (20.1)	
MPR (%)	99.5 (92.4–101.3)	99.9 (95.3–101.8)	60.9 (38.3–70.2)	N/A	<0.01*
Refills	8 (3–10)	8 (3–11)	7 (3–10)	N/A	0.10
Age, years	62.7 ± 9.1	63.1 ± 8.5	62.8 ± 10.7	61.3 ± 10.2	0.30
Male sex	230 (58.5)	168 (60.0)	20 (58.8)	42 (53.2)	0.55
Diabetes duration, years	11 [7–18]	12 [7–19]	9 [5–15]	9 [4–14]	<0.01*
BMI, kg/m^2^ ^a^	33.0 ± 6.2	33.2 ± 6.2	31.2 ± 6.8	32.9 ± 6.0	0.19
Smoking status					
Current	68 (17.3)	48 (17.1)	6 (17.6)	14 (17.7)	0.99
Former	209 (53.2)	153 (54.6)	19 (55.9)	37 (46.8)	0.45
Never	116 (29.5)	79 (28.2)	9 (26.5)	28 (35.4)	0.43
HbA1c, % (mmol/mol)^a^	7.4 ± 3,2 (57.0 ± 11.5)	7.4 ± 3.0 (57.0 ± 9.8)	7.8 ± 3.7 (61.5 ± 16.9)*	7.2 ± 3.4 (55.3 ± 13.7)*	0.031*
Serum cholesterol, mmol/L^a^	3.97 ± 0.90	3.72 ± 0.77	4.25 ± 1.00	4.72 ± 0.86	<0.01*
LDL cholesterol, mmol/L	1.99 ± 0.75	1.76 ± 0.60	2.23 ± 0.90	2.71 ± 0.67	<0.01*
LDL cholesterol ≤2.5 mmol/L	313 (79.6)	252 (90.0)	25 (73.5)	36 (45.6)	<0.01*
LDL cholesterol <1.8 mmol/L	172 (43.8)	158 (56.4)	9 (26.5)	5 (6.3)	<0.01*
Systolic BP, mmHg^a^	139 ± 16	139 ± 16	139 ± 15	141 ± 15	0.58
Diastolic BP, mmHg^a^	76 ± 9	75 ± 9*	76 ± 8	78 ± 9*	0.017*
Microvascular complications	271 (69.0)	202 (72.1)	25 (73.5)	44 (55.7)	0.017*
Neuropathy	140 (35.6)	104 (37.1)	10 (29.4)	26 (32.9)	0.58
Retinopathy^a^	92 (23.4)	71 (25.4)	10 (29.4)	11 (13.9)	0.09
DKD	158 (40.2)	121 (43.2)	15 (44.1)	22 (27.8)	0.049*
Macrovascular complications	142 (36.1)	109 (38.9)	15 (44.1)	18 (22.8)	0.018*
Insulin use	246 (62.6)	183 (65.4)	22 (64.7)	41 (51.9)	0.09
Antihypertensive drug use	323 (82.2)	243 (86.8)	26 (76.5)	54 (68.4)	<0.01*

Data are presented as *n* (%), mean ± SD, or median (interquartile range) for nominal, normally distributed, and nonnormally distributed data, respectively.

Abbreviations: MPR, Medication Possession Ratio; LDL, Low-Density Lipoprotein; DKD, Diabetic kidney disease.

a Missing values for BMI (*n* = 2), HbA1c (*n* = 2), serum cholesterol (*n* = 3), systolic blood pressure (*n* = 2), diastolic blood pressure (*n* = 2), retinopathy (*n* = 2), DKD (*n* = 2).

*Statistically significant difference between the groups (*p* value < 0.05).

### Medication Adherence

Of our total study population, 314 out of 393 (80%) patients had a statin prescription and of these, 280 (89%) were found to be adherent (Table 1). MPR rates for adherent and nonadherent patients were approximately 100% (95–102%) and 61% (38–70%), respectively. The adherent, nonadherent, and non-statin groups had a similar age, sex distribution, and BMI.

### Medication Adherence and Cholesterol Levels

**FIGURE 1:**
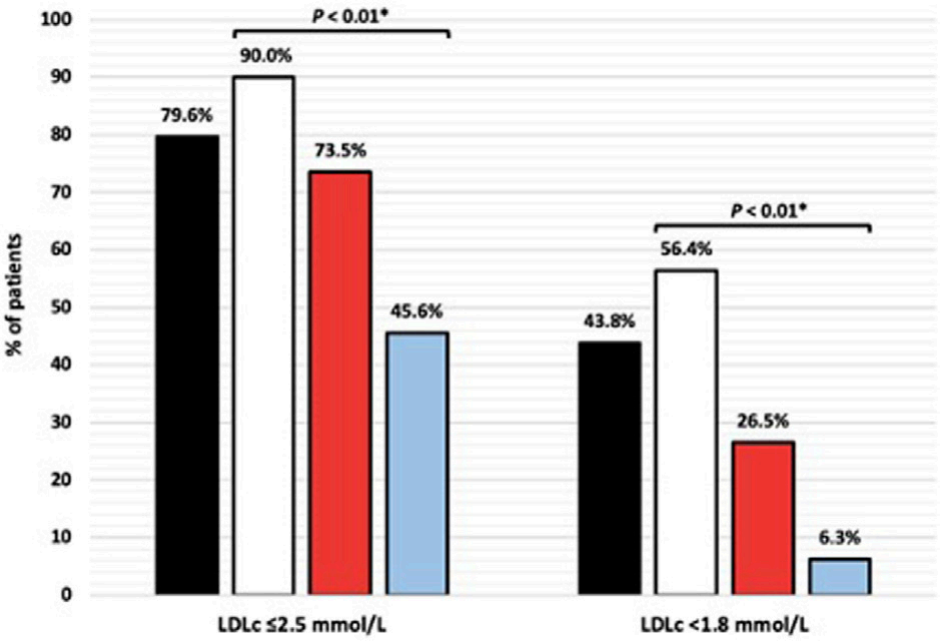
Attainment of LDL cholesterol (LDLc) targets by adherence. Black bars, total population; white bars, adherent; red bars, nonadherent; blue bars, no statin. *p < 0.05 indicates significant differences between adherent, nonadherent and non-statin groups.

The proportion of patients who reached the LDLc target of ≤2.5 mmol/L differed significantly between the adherent, nonadherent and non-statin groups (90% vs. 74% vs. 46%, respectively; *p* < 0.01) (Figure 1). The same applied to attainment of the LDLc target of <1.8 mmol/L (56% vs. 26.5% vs. 6%; *p* < 0.01). Accordingly, serum LDLc levels were significantly different between the adherent, nonadherent and non-statin groups (1.76 ± 0.60 vs. 2.23 ± 0.90 vs. 2.71 ± 0.67 mmol/L; *p* < 0.01) (Table 1). The same was true for to total cholesterol levels (3.72 ± 0.77 vs. 4.25 ± 1.00 vs. 4.72 ± 0.86 mmol/L; *p* < 0.01).

### Medication Adherence and Other Cardiovascular Risk Factors

Diabetes duration did not differ between adherent and nonadherent patients [12 (7–19) vs. 9 (5–15) years; *p* = 0.067], but was significantly higher in adherent patients compared with patients without statin prescription [12 (7–19) vs. 9 (4–14) years; *p* < 0.01)] (Table 1). HbA1c levels were significantly higher in nonadherent patients compared with adherent patients [61.5 ± 16.9 (7.8 ± 3.7%) vs. 57.0 ± 9.8 mmol/mol (7.4 ± 3.0%); *p* = 0.031]. No significant differences were found between systolic and diastolic blood pressure levels in the two groups using statins. However, diastolic blood pressure was significantly higher in patients without statin prescription compared with adherent patients (78 ± 9 vs. 75 ± 9 mmHg; *p* < 0.01). The proportion of patients with an insulin or antihypertensive drug prescription did not differ between adherent and nonadherent patients, but antihypertensive drug use was significantly lower in patients without statin prescription compared with adherent patients (68% vs. 87%; *p* < 0.01).

### Medication Adherence and Diabetes Complications

The prevalence of microvascular and macrovascular complications did not differ between adherent and nonadherent patients (72% vs. 74%; *p* = 0.87 and 39% vs. 44%; *p* = 0.56, respectively) (Table 1). However, the prevalence of these complications was significantly lower in patients without statin prescription compared with adherent and nonadherent patients (56%; *p* = 0.017 and 23%; *p* = 0.018). Within the individual components of microvascular complications, the prevalence of diabetic kidney disease was significantly lower in patients without statin prescription compared with adherent and nonadherent patients (28% vs. 43% vs. 44%, respectively; *p* = 0.049).

### Medication Adherence and Type and Intensity of Statin Therapy

Regarding statin prescriptions, the most common compound was simvastatin (49%) (Table 2). In the nonadherent group, the proportion of patients who had a prescription of simvastatin was significantly higher compared with adherent patients (68% vs. 47%, *p* = 0.024). No significant associations were found between adherence and other statin subtypes or treatment intensity.

**TABLE 2:** Statin subtype prescription and treatment intensity.

	Total population	Adherent	Nonadherent	*p*
Subtype				
Overall	314	280 (89.2)	34 (10.8)	
Simvastatin	155 (49.4)	132 (85.2)	23 (14.8)	0.024*
Atorvastatin	63 (20.1)	58 (92.1)	5 (7.9)	0.41
Rosuvastatin	72 (22.9)	67 (93.1)	5 (6.9)	0.23
Pravastatin	20 (6.4)	19 (95.0)	1 (5.0)	0.39
Fluvastatin	3 (1.0)	3 (100)	0 (0)	0.54
Treatment intensity				
Low	23 (7.3)	22 (95.7)	1 (4.3)	0.30
Medium	209 (66.6)	183 (87.6)	26 (12.4)	0.20
High	81 (25.8)	74 (91.4)	7 (8.6)	0.46

Data are presented as *n* (%).

*Statistically significant difference between the groups (*p* value < 0.05).

### Determinants of Nonadherence

Multivariate binary logistic regression (Table 3) indicated that higher HbA1c levels (OR: 1.05, 95% CI 1.01–1.08 per 1 mmol/mol increment in HbA1c; *p* < 0.01) were independently associated with nonadherence. No significant associations were found for diabetes duration, BMI, microvascular complications, macrovascular complications, insulin prescription, antihypertensive drug prescription, and statin prescription.

**TABLE 3:** Independent determinants of nonadherence to statins.

Variable	OR (95% CI) univariate	*p* value univariate	OR (95% CI) multivariate	*p* value multivariate
Diabetes duration	0.96 (0.91–1.00)	0.07	0.95 (0.89–1.00)	0.06
High BMI	0.94 (0.88–1.01)	0.07	0.94 (0.88–1.01)	0.10
HbA1c (mmol/mol)	1.04 (1.01–1.07)	0.024	1.05 (1.01–1.08)	<0.01
Microvascular complications	1.07 (0.48–2.40)	0.87	1.22 (0.49–3.01)	0.67
Macrovascular complications	1.24 (0.60–2.54)	0.56	1.83 (0.79–4.21)	0.16
Insulin prescription	0.97 (0.46–2.05)	0.94	1.30 (0.54–3.14)	0.56
Any antihypertensive treatment	0.50 (0.21–1.18)	0.11	2.45 (0.91–6.59)	0.08
Statin prescription				
Simvastatin	Ref.		Ref.	
Atorvastatin	0.50 (0.18–1.37)	0.17	0.42 (0.14–1.26)	0.12
Rosuvastatin	0.43 (0.16–1.18)	0.10	0.52 (0.18–1.51)	0.23
Pravastatin	0.29 (0.04–2.24)	0.23	0.35 (0.04–2.85)	0.32
Fluvastatin	^a^		^a^	

Fully adjusted logistic regression model with nonadherence as study outcome. Abbreviation: OR, odds ratio.

a Fluvastatin was prescribed in only three patients, which were all adherent These three patients were omitted for the purpose of analysis.

### Determinants of Attaining the LDLc Target of ≤2.5 mmol/L

Multivariate binary logistic regression (Table 4) indicated that medication adherence (OR: 2.88, 95% CI 1.04–7.97; *p* = 0.041), lower BMI (OR: 0.88, 95% CI 0.81–0.96 per 1 kg/m^2^ decrement in BMI; *p* < 0.01), and pravastatin prescription (OR: 11.53, 95% CI 3.69–36.01; *p* < 0.01) were independently associated with attaining the LDLc target of ≤2.5 mmol/L. No significant associations were found for diabetes duration, HbA1c, microvascular complications, macrovascular complications, insulin prescription, antihypertensive drug prescription, and prescription of other statin subtypes.

**TABLE 4:** Independent determinants of attaining the LDLc target of ≤2.5 mmol/L.

Variable	OR (95% CI) univariate	*p* value univariate	OR (95% CI) multivariate	*p* value multivariate
Medication adherence	3.24 (1.38–7.63)	<0.01	2.88 (1.04–7.97)	0.041
Diabetes duration	0.97 (0.94–1.00)	0.049	0.99 (0.94–1.05)	0.83
High BMI	0.94 (0.90–0.99)	0.014	0.88 (0.81–0.96)	<0.01
HbA1c (mmol/mol)	1.01 (0.99–1.03)	0.45	1.03 (0.99–1.06)	0.15
Microvascular complications	1.06 (0.62–1.82)	0.82	0.81 (0.33–1.95)	0.63
Macrovascular complications	1.08 (0.65–1.79)	0.78	1.12 (0.49–2.55)	0.80
Insulin prescription	0.67 (0.41–1.11)	0.12	0.65 (0.27–1.55)	0.33
Any antihypertensive treatment	0.69 (0.38–1.26)	0.22	1.19 (0.36–3.93)	0.78
Statin prescription				
Simvastatin	Ref.		Ref.	
Atorvastatin	0.79 (0.30–2.07)	0.63	1.04 (0.36–3.01)	0.94
Rosuvastatin	0.55 (0.20–1.53)	0.25	0.59 (0.18–1.99)	0.40
Pravastatin	6.23 (2.37–16.37)	<0.01	11.53 (3.69–36.01)	<0.01
Fluvastatin	3.74 (0.32–43.21)	0.29	4.11 (0.32–53.60)	0.28

Fully adjusted logistic regression model with LDLc ≤2.5 mmol/L as study outcome.

Abbreviations: BMI, body mass index; OR, odds ratio.

## Discussion

### Main Results

In this report, we present the assessment of adherence to statins in a real-life population with T2D patients managed in routine secondary care using pharmacy dispensing data. To our knowledge, this is one of the first studies to report adherence in the real-world setting by calculating the MPR using pharmacy dispensing data and report the association of adherence with sociodemographic data, medical characteristics, LDLc levels, and LDLc target attainment in T2D patients. Generally, statin adherence levels were relatively high compared with those seen in other studies (17.8–79.2%) (Hope et al., 2019), and adherence was associated with lower LDLc levels. In addition, LDLc targets were reached less frequently in nonadherent patients. This highlights the importance of identifying nonadherence, as it appears to be an important determinant of failure to reach LDLc targets. Despite extensive evidence of the effectiveness of lipid-lowering drugs, the share of non-statin users in our study was high (20.1%). The finding that many patients who failed to attain LDLc targets did not receive statin treatment offers an opportunity to improve diabetes care.

### Related Research

In a recent study by Fang et al. (2021), a cross-sectional analysis of data from adults with diabetes in the United States participating in the National Health and Nutrition Examination Survey (NHANES), national trends in diabetes treatment and risk-factor control from 1999 through 2018 were assessed. They found that the use of statin medication plateaued after 2010 at approximately 56%. Our study confirms and extends this finding by demonstrating that in a health setting with well-established insurance coverage still many patients do not receive statin treatment and that non-adherence to statins is one of the determinants why patients do not reach targets.

As a possible explanation for the high degree of medication adherence in our population, one might speculate that patients treated in secondary care may feel more urgency to adhere to their treatment in comparison to patients treated in primary care. This is supported by comparing our study results with the results of the study of Guglielmi et al. (2017), where nonadherence rates of respectively 39% and 45% after 3 and 6 months were seen in patients treated in primary care.

In terms of urgency, another possible explanation for the high degree of medication adherence in our total population might be that the high prevalence of microvascular and macrovascular complications in the DIALECT population motivates patients to take their medication. However, the degree of diabetes complications does not explain why one patient was adherent and another was not, as the prevalence of microvascular and macrovascular complications did not differ between adherent and nonadherent patients in our study.

Furthermore, the well-organized pharmacy service in Netherlands could be a factor that improves adherence by frequent personalized contact between pharmacy staff and patients and proactive medication deliveries. Of the DIALECT population, 26 patients were using an automated medication dispensing system. Although organized delivery does not guarantee actual medication intake, the overall results as based on the MPR are very much in line with our previous findings regarding medication adherence based on LC-MS/MS analysis of urine samples in the same patients (Beernink et al., 2021).

Our main finding, i.e., that statin adherence was related to LDLc target attainment, is in line with a previous study in 653 patients with T2D treated for dyslipidaemia in a managed care diabetes program (Parris et al., 2005). The percentage of the patients achieving an LDLc target of ≤2.5 mmol/L was lower in that study versus ours (44% vs. 80%). The same applied to the median MPR rates, namely 70% versus 99.5% in our study. The differences in MPR rates could be a possible explanation for the differences in LDLc target attainment. One might speculate that differences in statin subtype prescription could also play a role in explaining the LDLc results. However, the most frequently prescribed compound in the former study was atorvastatin, which is known to be more potent than simvastatin (Law et al., 2003).

Of note, the level of adherence in our study was in the same range as previously reported in a large cohort of coronary heart disease patients that assessed associations of adherence with LDLc, where an overall MPR of 79.8% was found (Chi et al., 2014). In that study, 85.8% reached the 2.5 mmol/L LDLc target and 32.4% had a LDLc value less than 1.8 mmol/L, the latter percentage being considerably lower compared with our study, which might be explained by differences in prevailing guidelines and/or the presence of the additional underlying condition diabetes.

Despite the high medication adherence rates, a previous DIALECT study (Gant et al., 2017) showed low adherence rates to general lifestyle and dietary guidelines. Although medication adherence rates are high, adherence in the broad sense is much worse. In this context, one could also wonder whether the high medication adherence rates we found are specific to statins or are a reflection of overall medication adherence to any type of drug.

Regarding the high rate of non-statin users in our population, a previous DIALECT study (Gant et al., 2018) showed that of the patients without a statin prescription, a third did not have a prescription due to previous side-effects, another third did not have an indication for lipid-lowering therapy and in a third of the patients no reason was recorded for not having a statin prescription in the electronic patient file. Probably, patients in the latter subgroup did not want to use a statin because of previously experienced side-effects or a poor perception of statins. The possibility that a strict indication for lipid-lowering therapy was missed by the physician is unlikely, as the DIALECT population consists of patients with a very high risk of cardiovascular diseases. Furthermore, these patients are treated by a very committed team of nephrologists, pharmacists and specialized nurses, who are all aware of the current treatment guidelines.

### Strengths and Limitations

A strength of this study is that it was performed in a real-world population and that patients were unaware that medication adherence would be analysed. Another strength of this study is that, in addition to the majority of other studies on this subject, we assessed associations between adherence and LDLc levels and LDLc target attainment. The eventual provision of medication outside the pharmacy (e.g., during hospitalization) was not taken into account in this study. This could be considered as a limitation. Changes in treatment during the follow-up period were not included in the analyses, which could also be considered a limitation. Changes to another statin or dosage were carefully documented in the database.

The MPR is an adequate and commonly used proxy for medication use in retrospective studies. However, a limitation of assessing medication adherence by calculating the MPR is that patients who filled their prescription only once or did not fill their prescription the first time are not included, since the MPR can only be calculated for patients with at least two dispenses. Because of the secondary care setting and the high medication adherence, the proxy approach is legitimate. Another limitation of the MPR is that collection of medication does not guarantee actual medication intake. Nonetheless, the pharmacy dispensing data aligned with our previous assessment of adherence based on liquid chromatography-tandem mass spectrometry (LC-MS/MS) analysis of urine samples (Beernink et al., 2021). In that study, we found an overall adherence rate of 89.3% to oral antidiabetics, antihypertensives, and statins. However, we should note that the LC-MS/MS method was not appropriate to gain a complete picture of adherence for statins, since it cannot detect simvastatin (i.e., the most widely used statin in Netherlands) (Nederland, Zorginstituut, 2008). A comparison (data not reported) between the LC-MS/MS data and MPR adherence data showed that 91.9% of the patients who were adherent to detectable statins based on LC-MS/MS were also adherent based on the MPR.

Finally, given the study design being observational, causality between adherence and study outcomes cannot be determined.

## Conclusion

Although nonadherence was only seen in a small proportion of the patients, it is important to recognize nonadherence early because nonadherent patients reach their LDLc targets much less often, putting them at risk for diabetes complications. In these patients, reasons for nonadherence should be explored, discussed, and personalized support should be provided. Additionally, we need to focus on identifying non-statin users at risk for complications and intensifying statin therapy to achieve better LDLc target attainment.

## Data Availability

The data that support the findings of this study are available from the corresponding authors upon reasonable request.
