# Supplementary material for: Adherence to Statin Therapy and Attainment of LDL Cholesterol Targets in an Outpatient Population of Type 2 Diabetes Patients: Analysis in the DIAbetes and LifEstyle Cohort Twente (DIALECT)
Source: Front Pharmacol. 2022 Jul 12;13:888110. doi: 10.3389/fphar.2022.888110 (PMC9315395; doi:10.3389/fphar.2022.888110)
Supplement: Supplementary file 1 [file Table1.DOCX]

**Supplemental table 1. Baseline characteristics of the 42 excluded patients.**

|  | **Total population** | **Statin prescription** | **No statin**  **prescription** |
| --- | --- | --- | --- |
| **Patients** | 42 | 29 (69.0) | 13 (31.0) |
| **Age, years** | 64.6 ± 7.5 | 65.5 ± 7.2 | 62.8 ± 8.0 |
| **Male sex** | 24 (57.1) | 15 (62.5) | 9 (37.5) |
| **Diabetes duration, years** | 11 [7-14] | 12 [8-15] | 8 [7-11] |
| **BMI (kg/m^2^)** | 33.1 ± 5.9 | 33.7 ± 6.1 | 31.7 ± 5.5 |
| **Smoking status** |  |  |  |
| Current | 6 (14.3) | 4 (13.8) | 2 (15.4) |
| Former | 19 (45.2) | 14 (48.3) | 5 (38.5) |
| Never | 17 (40.5) | 11 (37.9) | 6 (46.2) |
| **HbA1c, % (mmol/mol)** | 57.7 ± 11.0 | 57.5 ± 12.5 | 58.1 ± 6.9 |
| **Serum cholesterol, mmol/L** | 4.30 ± 1.11 | 3.98 ± 0.88 | 5.03 ± 1.24 |
| **LDL cholesterol, mmol/L^†^** | 2.16 ± 0.65 | 1.99 ± 0.61 | 2.55 ± 0.58 |
| **LDL cholesterol** ≤**2.5 mmol/L** | 20 (60.6) | 17 (73.9) | 3 (30.0) |
| **LDL cholesterol <1.8 mmol/L** | 11 (33.3) | 9 (39.1) | 2 (20.0) |
| **Systolic BP, mmHg** | 141 ± 20 | 141 ± 19 | 141 ± 24 |
| **Diastolic BP, mmHg** | 77 ± 13 | 76 ± 12 | 79 ± 14 |
| **Microvascular disease** | 33 (78.6) | 25 (86.2) | 8 (61.5) |
| Neuropathy | 17 (40.5) | 10 (34.5) | 7 (53.8) |
| Retinopathy | 12 (28.6) | 9 (31.0) | 3 (23.1) |
| DKD | 24 (57.1) | 21 (72.4) | 3 (23.1) |
| **Macrovascular disease** | 15 (35.7) | 11 (37.9) | 4 (30.8) |
| **Insulin use** | 26 (61.9) | 20 (69.0) | 6 (46.2) |
| **Antihypertensive drug use** | 35 (83.3) | 26 (89.7) | 9 (69.2) |

Data are presented as n (%), mean ± SD, or median [interquartile range] for nominal, normally distributed, and nonnormally distributed data, respectively.

Abbreviations: LDL, Low-Density Lipoprotein; DKD, Diabetic kidney disease.

**^†^** Missing values for LDL cholesterol (n= 9)
